# Evaluation of lipase levels in patients with nephropathia epidemica - no evidence for acute pancreatitis

**DOI:** 10.1186/s12879-015-1031-8

**Published:** 2015-07-25

**Authors:** Daniel Kitterer, Ferruh Artunc, Stephan Segerer, M. Dominik Alscher, Niko Braun, Joerg Latus

**Affiliations:** Department of Internal Medicine, Division of General Medicine and Nephrology, Robert-Bosch-Hospital, Auerbachstr. 110, 70376 Stuttgart, Germany; Department of Internal Medicine, Division of Endocrinology, Diabetology, Vascular Disease, Nephrology and Clinical Chemistry, University of Tuebingen, Tuebingen, Germany; Division of Nephrology, University Hospital, Zurich, Switzerland

**Keywords:** Hantavirus, Puumala virus, HFRS, Nephopathia epidemica, Pancreatitis, Lipase

## Abstract

**Background:**

The most common causative agent for hemorrhagic fever with renal syndrome in Germany is Puumala virus (PUUV) and a high percentage of patients with PUUV infection have gastrointestinal (GI) symptoms. The aim of the present study was to determine the prevalence of increased lipase levels and acute pancreatitis during nephropathia epidemica (NE) in 166 patients from Germany.

**Methods:**

Clinical and laboratory data during the acute phase of the disease were obtained from medical reports and files from 456 patients during acute hantavirus infection. Patients in whom serum lipase levels were determined during acute course of the disease were included in the study.

**Results:**

Lipase levels at the time of diagnosis were determined in 166 of the 456 NE patients (36 %). Of the 166 patients, 25 (15 %) had elevated lipase levels at the time of admission to hospital or first contact with general practitioner/nephrologist. In total 7 patients had a threefold increased serum lipase above the normal range. Abdominal pain was not more often present in the group of patients with elevated serum lipase compared to the lipase-negative group (9/25 *vs* 58/141). Abdominal ultrasound and CT scans revealed no signs of pancreatitis in any of the patients. Patients with elevated serum lipase had higher serum creatinine peak levels (*p* = 0.03) during the course of the disease.

**Conclusions:**

Elevated lipase levels were common in our patient cohort and might reflect a more severe form of NE. NE does not lead to acute pancreatitis.

## Background

Pathogenic hantaviruses are able to cause disease in humans and after inhalation of virus containing aerosols to known syndromes may arise: haemorrhagic fever with renal syndrome (HFRS), with the milder subtype nephropathia epidemica (NE) and the hantavirus cardiopulmonary syndrome (HCPS). HFRS is endemic in large parts of Eastern and Northern Europe and Asia, whereas clustered outbreaks of HCPS (with case fatality rates (up to 60 %) [1, 2]) have been reported in South and North America [2–4] The growing list of affected countries (developing and developed countries) has led to public health concerns, especially after the outbreak of hantavirus infection in the Yosemite National Park in California during the summer of 2012 and the incidence peak in Germany in 2012 [5, 6]. In Germany, the incidence of HFRS increased from 0.09 cases/100.000 persons in 2001 to 2.47 cases/100.000 persons in 2010 [7]. The most common causative agent for HFRS in Germany is Puumala virus (PUUV) [5]. PUUV causes nephropathia epidemica (NE) [8, 9] and patients typically present with acute kidney injury and thrombocytopenia [10, 11].

Within the prodromal phase of about 3–5 days with flu-like symptoms (fever, headache, nausea/vomiting and/or visual disturbances), a high percentage of patients with PUUV infection have severe gastrointestinal (GI) symptoms, e.g. abdominal pain, nausea and vomiting [9, 10, 12–16]. The clinical presentation might be misleading, resulting in surgery e.g. for suspected appendicitis. Some studies from Asia, Albania and the United States have reported that abdominal pain during the acute phase of HFRS may be caused by acute pancreatitis [17–21]. There have been two case reports of patients with acute pancreatitis during acute HFRS (total 3 patients) from Albania [21] and Korea [20], and further studies from Asia [17, 19] and the United States [18] revealed 25 of 387 patients (6.5 %) with acute pancreatitis during HFRS. In these studies, the acute pancreatitis rate during the acute hantavirus infection ranged between 2.8 % and 54 %. Differential diagnosis of acute kidney injury with signs of systemic inflammation is broad including e.g. leptospirosis (Weil’s disease, Stuttgart disease), sepsis, autoimmune disease, and thrombotic microangiopathy. Recently, we could show that procalcitonin could not be used to exclude acute NE [22]. Therefore, laboratory testing in the emergency department or at first contact with general practitioner/nephrologist include mainly various laboratory values.

It is noteworthy that serum lipase has a sensitivity and specificity for acute pancreatitis ranging from 82 to 100 % [23]. Additionally, increased levels of pancreatic enzymes have been reported in patients with impaired renal function even in the absence of pancreatic diseases although lipase tended to be less frequently raised than pancreatic enzymes [24]

To date, the prevalence of elevated lipase levels and acute pancreatitis and its impact on the clinical course of the disease has not been studied in a cohort of patients with PUUV-induced NE.

The aim of the present study was to determine the prevalence of increased lipase levels and acute pancreatitis during NE in 166 patients from Germany.

## Methods

### Patients

Between 2001 and 2012, 7476 patients with serologically and clinically confirmed NE were reported to the Robert Koch Institute in Berlin (Robert Koch Institute, SurvStat, www3.rki.de/SurvStat). All patients met the national case definition for hantavirus infection [25, 26] In cooperation with four selected local health authorities in southern Germany (Stuttgart, Boeblingen/Sindelfingen, Esslingen, Reutlingen), all infected patients between 2001 and 2012 were contacted via mail requesting an appointment in our outpatient clinic (in total, 1570 patients were serologically confirmed to have NE).

Between September 2012 and April 2013, 456 out of the 1570 contacted patients with serologically and clinically confirmed NE were included in our study. All patients gave written consent before participating in the study, which was approved by the Ethics Committee of the Ethics Commission of the State Chamber of Medicine in Baden-Württemberg (Stuttgart) (F-2012-046). Studies were conducted in concordance with the Declaration of Helsinki.

### Data acquisition

#### Acute phase of NE

Clinical and laboratory data at the time of diagnosis and during the acute course of the disease were obtained from medical reports and files from the 456 patients. Patients in whom lipase levels were measured during acute course of the disease were included in the current analysis. Patients with serum lipase levels ≥ 60 U/L (normal range < 60 U/L in our laboratory) were classified as lipase positive, while those with serum lipase levels < 60 U/L were classified as lipase negative. Lipase levels were not measured routinely in our study population, but rather at the treating physician’s discretion (certainly in cases of suspected acute pancreatitis).

#### Definition of diagnosis of acute pancreatitis

The diagnosis of acute pancreatitis requires two of the following three features: (1) abdominal pain consistent with acute pancreatitis (acute onset of a persistent, severe, epigastric pain often radiating to the back); (2) serum lipase activity (or amylase activity) at least three times greater than the upper limit of normal; and (3) characteristic findings of acute pancreatitis on contrast-enhanced computed tomography (CECT) and less commonly magnetic resonance imaging (MRI) or transabdominal ultrasonography [27–29].

#### Statistical analysis

All continuous variables were tested for normal distribution using the Kolmogorov–Smirnov test and are presented as means ± standard deviations. The median with interquartile range is reported where the distribution was not normal. Nonparametric tests (Fisher’s exact test, Mann–Whitney *U* test) were used for statistical analysis.

## Results

Lipase levels were determined in 166 of the total 456 (36 %) patients at the time of diagnosis of NE. The baseline characteristics of the study population are shown in Table 1. Of the 166 patients, 141 (85 %) had lipase levels within the normal range (<60 U/L) at time of admission to hospital or first contact with general practitioner/nephrologist. Conversely, 25 of the 166 patients had elevated lipase levels (≥60 U/L) and 28 % of these patients had a threefold increased serum lipase above the normal range (7 patients, Table 2). In total, 67 of the 166 patients (40 %) had abdominal pain at time of diagnosis. The number of patients with increased lipase levels was not significantly different between the groups with and without abdominal pain (9/25 *vs* 58/141). Abdominal ultrasound was performed in 88 % of the patients with elevated serum lipase, but demonstrated no signs of acute pancreatitis. In two patients, CT scans of the abdomen were performed, revealing no signs of pancreatitis.

**Table 1:** Clinical data of 166 patients in whom serum lipase was measured at time of admission to hospital or first contact with general practitioner/nephrologist, significant *p* values were marked in bold

Variable	Patients with normal serum lipase	Patients with elevated serum lipase	*p*
*n*	141	25	-
Age (years ± SD)	45.3 ± 14.2	57.7 ± 12.2	**0.001**
Female/male	56/85	6/19	0.798
Onset of symptoms prior to admission to hospital (days)	5.5 (3.6-7.0)	4.5 (3.8-7.3)	0.398
Duration of hospital stay (days)	7.0 (4.0-9.0)	6.5 (5.0-11.0)	0.694
**Symptoms**			
Abdominal pain	58/141	9/25	1.000
Back-/flank pain	102/141	17/25	1.000
Headache	96/141	12/25	0.185
Visual disorders	35/141	6/25	0.566
Diarrhea	35/141	7/25	1.000
Nausea/vomiting	69/141	10/25	1.000
**Laboratory findings**			
Thrombocytes at admission (×10^9^/L)	105.5 (78.0-172.5)	91.0 (73.5-215.5)	0.233
Minimum thrombocyte level (×10^9^/L)	98.0 (69.8-161.5)	91.0 (73.0-207.5)	0.167
Creatinine at admission (mg/dL [0.5-1.4])	1.7 (1.1-3.5)	2.9 (1.6-5.1)	**0.008**
Creatinine peak level (mg/dL [0.5-1.4])	2.7 (1.7-4.6)	4.6 (2.7-6.8)	**0.025**
CrP at admission (mg/dL [0.1-0.4])	4.1 (2.9-7.5)	5.8 (2.5-8.5)	0.871
CrP peak level (mg/dL [0.1-0.4])	4.8 (3.0-8.5)	5.6 (2.4-8.8)	0.792
Lactate dehydrogenase, serum	273.5 (242.0-318.0)	285.0 (239.0-374.5)	0.348
**Clinical signs**			
Fever	123/141	21/25	0.280
Blood pressure			
systolic	130.0 (119.0–140.0)	147.0 (124.8-157.8)	**0.010**
diastolic	80.0 (70.0–89.0)	80.0 (74.9-97.5)	0.306
Heart rate	76.5 (63.0–88.0)	80.5 (75.5-91.8)	0.471
Abdominal ultrasound	119/141	22/25	0.770
Abdominal CT scan, native	5/141	3/25	0.101

**Table 2:** Clinical data of 25 patients with elevated Lipase and acute PUUV infection, significant *p* values were marked in bold

Variable	Patients with elevated serum lipase within threefold normal	Patients with lipase at least three times greater than the upper limit	
*n*	18	7	
Age (years ± SD)	58 ± 12.8	56 ± 11.3	
Female/male	7/11	1/6	
**Symptoms**			
Abdominal pain	7/18	2/7	
Back-/flank pain	13/18	4/7	
Headache	9/18	3/7	
Visual disorders	3/18	3/7	
Diarrhea	4/18	3/7	
Nausea/vomiting	9/19	1/7	
**Laboratory findings**			
Thrombocytes at admission (×10^9^/L)	117 (75–276)	82 (59–111)	
Creatinine at admission (mg/dL [0.5-1.4])	3.7 (1.7-6.2)	2.5 (1.9-2.9)	
Creatinine peak level (mg/dL [0.5-1.4])	5.6 (2.9-6.9)	3.1 (2.7-4.5)	
CrP at admission (mg/dL [0.1-0.4])	5.4 (2–6.8)	8.0 (4.6-8.7)	
Lactate dehydrogenase, serum	285.0 (229–334)	313.0 (376–256)	
**Clinical signs**			
Fever	14/18	7/7	
Blood pressure mmHg			
Systolic mmHg	150 (140–160)	120 (100–150)	
Diastolic mmHg	86 (75–96)	80.0 (72–91)	

Patients with elevated serum lipase were significantly older (*p* = 0.01), had higher serum creatinine levels at admission (*p* = 0.008), and higher serum creatinine peak levels (*p* = 0.03) throughout the course of the disease.

Duration of hospital stay was not different between both groups (*p* = 0.7). Neither C-reactive protein (CRP) levels nor thrombocyte counts were different between both groups at time of admission to hospital or first contact with general practitioner/nephrologist and throughout the course of the disease (*p* > 0.05). Patients with at least threefold increased serum lipase above the normal range had lower creatinine levels (2.5 (1.9-2.9) mg/dl *vs* 3.7 (1.7-6.2) mg/dl), but lower thrombocyte counts (117 (75–277) × 10^9^/L *vs* 82 (59–11) × 10^9^/L) at time of admission to hospital or first contact with general practitioner/nephrologist and lower creatinine peak levels during acute course of the disease (3.1 (2.7-4.5) mg/dl *vs* 5.6 (2.9-6.9) mg/dl) without reaching statistical significance (all *p* > 0.5) (Table 2).

We compared baseline characteristics (e.g. symptoms, laboratory findings, age, creatinine, CRP levels at hospital admission) during the acute course of the disease between patients in whom lipase levels had been tested (*n* = 166) and those in whom lipase had not been tested (*n* = 290). No statistically significant differences were observed between the groups except that, in the group of patients in whom lipase levels had been determined, there was a higher number of patients with abdominal pain (*p* = 0.02), with a lower thrombocyte count (*p* < 0.05), presenting with headache (*p* < 0.001), and in whom abdominal ultrasound and CT scans had been performed (Table 3).

**Table 3:** Clinical data of 456 patients with PUUV infection, significant *p* values were marked in bold

Variable	Patients in whom lipase was determined	Patients in whom lipase was not determined	*P*
*n*	166	290	-
Age (years ± SD)	47.4 ± 14.6	48.8 ± 14.6	0.249
Female/male	63/103	102/188	0.613
Onset of symptoms prior to admission to hospital (days)	5.0 (3.6-7.0)	5.0 (3.0-7.0)	0.080
Duration of hospital stay (days)	7.0 (4.0-10.0)	7.0 (5.0-9.0)	0.999
**Symptoms**			
Abdominal pain	67/166	84/290	**0.017**
Back-/flank pain	119/166	188/290	0.147
Headache	108/166	139/290	**0.0004**
Visual disorders	41/166	60/290	0.349
Diarrhea	42/166	50/290	0.052
Nausea/vomiting	79/166	136/290	0.923
**Laboratory findings**			
Thrombocytes at admission (×10^9^/L)	105.0 (76.5-174)	128.5 (87.8-213.5)	**0.005**
Minimum thrombocyte level (×10^9^/L)	96.0 (71.0-163.0)	124 (77.8-212.0)	**0.007**
Creatinine at admission (mg/dL [0.5-1.4])	1.8 (1.2-3.6)	1.8 (1.2-3.3)	0.983
Creatinine peak level (mg/dL [0.5-1.4])	3.0 (1.7-5.2)	2.7 (1.6-4.6)	0.218
CrP at admission (mg/dL [0.1-0.4])	4.4 (2.8-7.6)	3.9 (2.2-6.8)	0.094
CrP peak level (mg/dL [0.1-0.4])	5.0 (3.0-8.7)	4.5 (2.8-8.0)	0.340
Lactate dehydrogenase, serum	275.5 (247.0-327.0)	271.5 (240–324.5)	0.705
**Clinical signs**			
Fever	144/166	265/290	0.149
Blood pressure			
systolic	130.0 (119.0–146.0)	130.0 (120.0-146.0)	0.788
diastolic	80.0 (70.0–90.0)	80.0 (71.5-89.0)	0.951
Heart rate	77.6 (64.0–88.0)	74.5 (65.0-84.0)	0.148
Abdominal ultrasound	138/166	160/290	**<0.0001**
Abdominal CT scan	8/166	4/290	**0.035**

## Discussion

This is the first study to determine the prevalence of increased lipase levels and acute pancreatitis in a large cohort of NE patients. At time of diagnosis, a high percentage of patients with PUUV infection had severe gastrointestinal symptoms, e.g. abdominal pain, nausea and vomiting [9, 10, 12–16]. In one-third of our patient cohort, lipase levels were tested, which underlines that acute pancreatitis was a differential diagnosis for NE in patients with signs of inflammation, acute kidney injury with thrombocytopenia, and abdominal pain in a high proportion of patients. In our patient cohort, 15 % of the patients had elevated serum lipase levels at time of diagnosis and 7 patients had a threefold increased serum lipase above the normal range. Only two of them had abdominal pain and none of the patients had signs of acute pancreatitis using imaging techniques (abdominal ultrasound or CT scans). Other studies reported acute pancreatitis rates of between 2.8 % and 54 % during the course of acute hantavirus infection. Recently we demonstrated PUUV antigen in the appendix of an NE patient with severe acute kidney injury, who had undergone surgery because of severe abdominal pain. We investigated biopsies of the human intestine in patients with acute PUUV infection in order to understand why a majority of patients with NE have severe abdominal pain [15], for which there has so far been no pathophysiologic explanation. Immunohistochemical analysis revealed PUUV nucleocapsid antigen in 62 % of the analyzed biopsies, but the presence of PUUV antigen did not correlate with the extent of abdominal complaints [15]. Therefore, we can find no definitive explanation for the abdominal pain. PUUV nucleocapsid antigen was located mainly in endothelial cells of capillaries or larger vessels in the lamina propria of the biopsies of the human intestine. We hypothesized that systemic infection could be linked to the presence of PUUV nucleocapsid antigen in human intestine and might explain the clinical phenotype of severe gastrointestinal symptoms. At present, the cause of the abdominal symptoms is unknown. Involvement of the pancreas might be present in a significant part of the patients, as described in our cohort with about 15 %. This of course does not represent the sole factor of abdominal pain and there seem to be a group of patients with subclinical increases in lipase.

In our study population, only two out of seven patients presented with threefold increased serum lipase levels above the normal range. Remarkably, none of these patients received pancreatitis-specific treatment and none of the patients had an increase of lipase levels within the course of the disease.

Regarding differential diagnosis at time of first contact with the patient, it has to be mentioned that there is still a discussion whether pancreatic enzymes were elevated within threefold normal in patients with impaired kidney function [30, 31] and most NE patients still had acute kidney injury at time of diagnosis. Slightly elevated levels of lipase has been reported in patients with impaired renal function even in the absence of pancreatic diseases [24], but it is noteworthy that the 7 patients with lipase levels more than threefold normal in our study had less impaired clearance as compared to the other lipase positive patients. Therefore impaired clearance may not be the reason for the marked elevated lipase.

In these patients, elevated lipase levels should no lead to misdiagnose acute NE, but acute pancreatitis must be considered if enzyme levels are more than threefold normal in association with clinical manifestations.

In our present study, creatinine peak levels were significantly higher in the group with increased lipase levels compared to the group with normal lipase levels. This indicates a more severe course of the disease. Therefore, another possible explanation for elevated lipase levels in this group of patients could be an enhanced inflammatory response caused by a generalized PUUV infection involving endothelial cells of capillaries within the pancreas.

Several limitations of the evaluation need to be discussed. In our cohort of patients, lipase levels were measured at the physician’s discretion instead of a standardized manner (certainly in cases of suspected acute pancreatitis), which leads to a selection bias. Therefore the incidence of subclinical elevation of lipase levels might be higher. Furthermore, we performed a retrospective non-blinded study of medical case reports, with all known associated limitations, e.g. it would be quite helpful to have alternative biomarkers such as amylase or elastase measured in stool of the patients. Furthermore, amylase was not determined in our study population.

## Conclusions

This is the first study to investigate the prevalence of elevated lipase levels and acute pancreatitis and its impact on the clinical course in a large and representative cohort of patients with acute PUUV-induced NE. Elevated lipase levels are common in NE and may reflect a more severe form of NE. Slightly elevated lipase levels should no lead to misdiagnose acute NE, but acute pancreatitis must be considered if enzyme levels are more than threefold normal in association with clinical manifestations.
